# Molecular property prediction using pretrained-BERT and Bayesian active learning: a data-efficient approach to drug design

**DOI:** 10.1186/s13321-025-00986-6

**Published:** 2025-04-23

**Authors:** Muhammad Arslan Masood, Samuel Kaski, Tianyu Cui

**Affiliations:** 1 Department of Computer Science, Aalto University, Espoo, Finland; 2 Department of Computer Science, University of Manchester, Manchester, UK

**Keywords:** Drug discovery, Active learning, Bayesian, BERT

## Abstract

**Abstract:**

In drug discovery, prioritizing compounds for experimental testing is a critical task that can be optimized through active learning by strategically selecting informative molecules. Active learning typically trains models on labeled examples alone, while unlabeled data is only used for acquisition. This fully supervised approach neglects valuable information present in unlabeled molecular data, impairing both predictive performance and the molecule selection process. We address this limitation by integrating a transformer-based BERT model, pretrained on 1.26 million compounds, into the active learning pipeline. This effectively disentangles representation learning and uncertainty estimation, leading to more reliable molecule selection. Experiments on Tox21 and ClinTox datasets demonstrate that our approach achieves equivalent toxic compound identification with 50% fewer iterations compared to conventional active learning. Analysis reveals that pretrained BERT representations generate a structured embedding space enabling reliable uncertainty estimation despite limited labeled data, confirmed through Expected Calibration Error measurements. This work establishes that combining pretrained molecular representations with active learning significantly improves both model performance and acquisition efficiency in drug discovery, providing a scalable framework for compound prioritization.

**Scientific Contribution:**

We demonstrate that high-quality molecular representations fundamentally determine active learning success in drug discovery, outweighing acquisition strategy selection. We provide a framework that integrates pretrained transformer models with Bayesian active learning to separate representation learning from uncertainty estimation—a critical distinction in low-data scenarios. This approach establishes a foundation for more efficient screening workflows across diverse pharmaceutical applications.

## Introduction

Active learning (AL) is a semi-supervised machine learning approach that selects new data points to be labeled in an iterative process. Starting with a small initial dataset, the model strategically identifies and requests labels for the most informative samples from a larger unlabeled pool. These newly labeled points are then incorporated into the training set, and the model is retrained, progressively improving its predictive accuracy through each iteration[1]. This iterative approach enables efficient model development with minimal labeled data, making it particularly valuable when labeling is expensive or time-consuming.

In drug discovery, AL has become instrumental in efficiently identifying potent inhibitors from large molecular libraries, particularly in high-throughput screening (HTS) where exhaustive search is infeasible [2]. This approach allows for the efficient exploration of chemical space, targeting areas with the highest potential for success while maintaining structural novelty [3]. In drug property optimization, AL has significantly enhanced the prediction of pharmacokinetic parameters, yielding superior accuracy in rat plasma concentration predictions while utilizing only a fraction of conventional training data requirements [4]. The application of AL extends further into molecular design, where it has proven instrumental in guiding the exploration of chemical space and identifying compounds with desired physicochemical and biological properties. Notable achievements include the use of AL algorithms to direct generative models in synthesizing molecules that share structural similarities with known inhibitors, accomplishing this without prior exposure to the target compounds’ structures [5]. Recent innovations have explored the integration of human expertise with AL frameworks, establishing a novel paradigm for guiding molecular generation towards promising drug candidates [6].

The effectiveness of AL critically depends on accurate uncertainty estimation to guide the selection of informative training samples. Traditional approaches to uncertainty quantification in drug discovery have relied on various non-Bayesian methods. These include measuring distances in molecular descriptor space between input compounds and training sets [7, 8], or employing ensemble-based techniques where multiple model variants generate predictions to quantify uncertainty through their observed variance [9]. Predictive modeling is influenced by two fundamental uncertainty types: epistemic uncertainty, arising from insufficient data coverage in chemical space, and aleatoric uncertainty, stemming from experimental measurement noise [10]. While traditional methods such as training set distance metrics and ensemble model variance only capture epistemic uncertainty, auxiliary neural networks are required to quantify aleatoric uncertainty through the estimation of prediction variance [11]. However, Bayesian statistical frameworks offer a unified approach that captures both uncertainty types in a principled manner [12, 13].

Bayesian experimental design (BeD) formalizes the selection process by modeling uncertainties in predictions and using them to guide experimental choices. Several Bayesian acquisition functions have been developed to optimize the selection process. Bayesian Active Learning by Disagreement (BALD) selects samples that maximize information gain about model parameters [14], while Expected Predictive Information Gain (EPIG) prioritizes samples expected to most improve predictive performance [15].

Traditional quantitative structure–property relationships (QSPR) workflows rely on handcrafted descriptors that map 2D or 3D molecular structures into numerical vectors [16]. Modern deep learning approaches, particularly graph neural networks and transformer-based architectures, have transformed this paradigm by learning optimal structure-to-descriptor mappings directly from data, achieving superior performance over classical methods [16, 17]. However, these powerful neural networks cannot be directly applied in AL scenarios that typically begin with limited data ($\approx$100 molecules), as they tend to overfit the training data, leading to poorly calibrated uncertainty estimates that compromise the AL cycle’s effectiveness [18]. Semi-supervised learning addresses this challenge by leveraging unlabeled molecular datasets to pre-train neural networks [13, 19]. We extend this approach by integrating MolBERT [20], an adaptation of BERT [21] into the AL pipeline. This transformer-based BERT model, pretrained on 1.26 million compounds, allowed us to leverage a significant volume of unlabeled data, and encapsulated the contextual information of a larger chemical space. This integration enables robust uncertainty estimation with limited labeled data, bridging the gap between deep learning capabilities and AL constraints in drug discovery. Recently, Cao et al. [22] demonstrated that pretrained models can enhance sample efficiency in virtual screening; however, their Bayesian optimization approach focuses on identifying optimal compound that maximize specific property, while our active learning methodology selects informative datapoints to improve overall model performance.

## Materials and methods

### Datasets

*Tox21:* The Tox21 dataset, or Toxicology in the 21st Century dataset, is a publicly available dataset used in the field of computational toxicology [23]. The Tox21 dataset consists of a large collection of chemical compounds, each of which is associated with various types of toxicity outcomes. These outcomes are typically measured using high-throughput screening assays to evaluate the potential toxic effects of the compounds. The dataset provides a quantitative assessment (in form of binary labels) of toxicity of $\approx$ 8000 compounds in 12 different toxicity pathways. The Tox21 dataset is widely used as a benchmark in the development of in silico toxicology models. In this dataset, 6.24% measurements are active (ranges from 2% to 12%), 73% are inactive, while 20.56% are missing values.

*ClinTox:* The ClinTox dataset [24] combines data from two distinct sources: FDA-approved drugs and drugs that failed clinical trials due to toxicity. It contains information for 1,484 compounds with binary labels. The dataset provides valuable insights into the relationship between chemical structures and drug safety profiles in human clinical trials.

### Data splitting

*Test, train set:* For the better of evaluation of generalization, we employed scaffold splitting with 80:20 ratio to create distinct training and testing sets. Scaffold splitting partitions a molecular dataset according to core structural motifs identified by the Bemis-Murcko scaffold representation [25], prioritizing larger groups while ensuring that the train and test sets do not share identical scaffolds. The test set is identical for all the experiments.

*Initial and pool sets:* A balanced initial set was constructed by randomly selecting 100 molecules from the training set, with equal representation of positive and negative instances. Subsequently, a pool set was generated by excluding the initial set from the training set.

### Bayesian experimental design and active learning

Bayesian experimental design is a well-principled framework for quantifying the utility of conducting an experiment [26]. Specifically, let $\xi \in \varXi$ be the design that we would like to optimize in the design space $\varXi$ and *y* be the output of the experiment given the current design $\xi$ with a likelihood function $p(y|\xi )$. We would like to optimize an **acquisition function**, which is the expected utility function $U(\xi ,y)$ under $p(y|\xi )$,

1 $$\begin{aligned} \xi ^{\star }=\mathop {\mathrm {arg\,max}}\limits _{\xi \in \varXi }\mathbb {E}_{y\sim p(y|\xi )}\left[ U(\xi ,y)\right] , \end{aligned}$$

as *y* is unobserved before the experiment.

Active Learning is an application of experimental design to improve the labeling process. We consider fully supervised learning tasks, e.g., predicting molecular properties, using a probabilistic model with likelihood function $p(y|\varvec{x}, \phi )$, where $\varvec{x}$ is an input (a molecule), *y* is the output (molecular property), and $\phi$ is the parameter of the model $f(\varvec{x}; \phi )$ with a prior distribution $p(\phi )$. The corresponding posterior $p(\phi |\mathcal {D})$ given a labeled training set $\mathcal {D}=\{(\varvec{x}_i, y_i)\}_{i=1}^{N}$ can be obtained by Bayes’ rule: $p(\phi |\mathcal {D})\propto \prod _i^Np(y_i|\varvec{x}_i, \phi )p(\phi )$.

In AL or experimental design [26], we have access to another unlabeled set $\mathcal {D}_u=\{(\varvec{x}_i^u)\}_{i=1}^{N_u}$ (the design space $\varXi$) and would like to select the most **informative** (measured by the acquisition function) unlabeled data $\varvec{x}_s$ (the optimal design $\xi$) to label. By incorporating the new labeled data $(\varvec{x}_s^u, y_s)$ into the training set $\mathcal {D}=\mathcal {D}\bigcup \{(\varvec{x}_s^u, y_s)\}$, we have an improved posterior $p(\phi |\mathcal {D})$.

The informativeness of unlabeled data points is defined by the acquisition function. Two popular acquisition functions are given below:

*BALD Acquisition Function:* One popular acquisition function is Bayesian Active Learning by Disagreement (BALD) [14], which is the expected information gain, measured by the reduction in Shannon entropy of the model parameter $\phi$ from labeling $\varvec{x}$ across all possible realizations of its label *y* given by $p(y|\varvec{x},\mathcal {D})$. Specifically, we have $\text {BALD}(\varvec{x})=\mathbb {E}_{y\sim p(y|\varvec{x}, \mathcal {D})}\left[ {\textrm{H}}[\phi |\mathcal {D}]-{\textrm{H}}[\phi |\varvec{x},y,\mathcal {D}]\right]$, which is usually intractable due to the high-dimensional posterior over the parameters. By observing the equivalence between BALD and the conditional mutual information between the parameter and the unknown output, $\textrm{I}[\phi ,y|\varvec{x},\mathcal {D}]$, BALD can be rearranged to compute the information in the output space:

2 $$\begin{aligned} \begin{aligned} \text {BALD}(\varvec{x})&=\textrm{I}[\phi ,y|\varvec{x},\mathcal {D}]={\textrm{H}}[y|\varvec{x},\mathcal {D}]-\mathbb {E}_{\phi \sim p(\phi |\mathcal {D})}\left[ {\textrm{H}}[y|\varvec{x},\phi ]\right] \end{aligned} \end{aligned}$$

with the optimal design $\varvec{x}^{\star }=\mathop {\text{arg}\;\text{max}}_{\varvec{x}}\text {BALD}(\varvec{x})$. The first term in BALD measures the total uncertainty on the output *y* for its input $\varvec{x}$ while the second term measures its aleatoric uncertainty, i.e., the irreducible uncertainty from observational noise. Therefore, BALD selects $\varvec{x}$ with the highest epistemic uncertainty, i.e., the reducible uncertainty from the lack of data [27].

*EPIG Acquisition Function:* BALD targets global uncertainty reduction on the parameter space $\phi$. However, in most supervised learning tasks, users are interested in improving the model accuracy on a target set $p(\varvec{x}_*)$, e.g., the test set. Therefore, recent work [15] claimed that as acquisition function, Expected Predictive Information Gain (EPIG), explicitly reducing the model output uncertainty on random samples from $p(\varvec{x}_*)$ is more effective than BALD in improving the model performance, defined as:

3 $$\begin{aligned} \begin{aligned} \text {EPIG}(\varvec{x}) = \mathbb {E}_{p(\varvec{x}_*)}\left[ {\textrm{H}}[y_*|\varvec{x}_*, \mathcal {D}]-\mathbb {E}_{p(y|\varvec{x},\mathcal {D})}\left[ {\textrm{H}}[y_*|\varvec{x}_*, y, \varvec{x}]\right] \right] \end{aligned} \end{aligned}$$

is *expected* reduction of the “expected predictive uncertainty” over the *target input distribution* $p(\varvec{x}_*)$ by observing the label of $\varvec{x}$. Intuitively, compared with BALD which reduces the parameter uncertainty globally, EPIG only reduces the parameter uncertainty that reduces model output uncertainty on $p(\varvec{x}_*)$.

*Approximating acquisition functions:* In practice, the posterior $p(\phi |\mathcal {D})$ is intractable, but we can approximate each of the acquisition functions using an approximated distribution $q(\phi )$, such as the dropout distribution [28] used in Section 2.5. Specifically, for BALD, the acquisition function can be rewritten as:

4 $$\begin{aligned} \begin{aligned} \text {BALD}(\varvec{x})&={\textrm{H}}[y|\varvec{x},\mathcal {D}]-\mathbb {E}_{\phi \sim p(\phi |\mathcal {D})}\left[ {\textrm{H}}[y|\varvec{x},\phi ]\right] \\&=-\sum _{c\in \{0,1\}}p(y=c|\varvec{x},\mathcal {D})\log p(y=c|\varvec{x},\mathcal {D})+\mathbb {E}_{q(\phi )}\left[ \sum _{c\in \{0,1\}}p(y=c|\varvec{x},\phi )\log p(y=c|\varvec{x},\phi )\right] , \end{aligned} \end{aligned}$$

where *c* is the class label that *y* can take and $p(y=c|\varvec{x},\mathcal {D})\approx \mathbb {E}_{q(\phi )}\left[ p(y=c|\varvec{x},\phi )\right]$.

For EPIG [15], first we observe

5 $$\begin{aligned} \begin{aligned} \text {EPIG}(\varvec{x})&=\mathbb {E}_{p(\varvec{x}_*)}\left[ \textrm{KL}\left[ p(y,y_*|\varvec{x},\varvec{x}_*,\mathcal {D})|p(y|\varvec{x},\mathcal {D})p(y_*|\varvec{x}_*,\mathcal {D})\right] \right] , \end{aligned} \end{aligned}$$

where $$p(y|\varvec{x},\mathcal {D})\approx \mathbb {E}_{q(\phi )}\left[ p(y|\varvec{x}, \phi )\right] $$ and $$p(y,y_*|\varvec{x},\varvec{x}_*,\mathcal {D})\approx \mathbb {E}_{q(\phi )}\left[ p(y|\varvec{x}, \phi )p(y_*|\varvec{x}_*, \phi )\right] $$.

All expectations in above acquisition functions can be approximated with Monte Carlo sampling. For example, with *T* samples from $q(\phi )$:

6 $$\begin{aligned} \begin{aligned} \mathbb {E}_{q(\phi )}\left[ p(y|\varvec{x}, \phi )\right] \approx \frac{1}{T}\sum _{t=1}^{T}p(y|\varvec{x}, \phi ^{(t)}), \end{aligned} \end{aligned}$$

where $$\phi ^{(t)}\sim q(\phi )$$.

*Uniform (Random) Acquisition Function:* The uniform(random) acquisition function randomly selects unlabeled data points with equal probability, serving as a baseline strategy. Specifically, for any unlabeled input $\varvec{x}\in \mathcal {D}$, the uniform acquisition function is defined as:

7 $$\begin{aligned} \begin{aligned} \text {UNIFORM}(\varvec{x}) = \frac{1}{|\mathcal {D}|}, \end{aligned} \end{aligned}$$

where $|\mathcal {D}|$ is the size of the pool dataset. While simple, this strategy provides an important baseline for comparing more sophisticated acquisition functions like BALD and EPIG, as it helps quantify the benefits of AL over random sampling.

### Semi-supervised active learning (SSAL)

In the fully supervised scenario, the model $f(\varvec{x};\phi )$ only learns from the labelled dataset $\mathcal {D}$. This is inefficient in AL because the labelled dataset for training is limited initially, and AL has to collect more data to learn a good input manifold, which is required to estimate the uncertainty of downstream tasks [19]. This is particularly challenging in the chemical space, where the input manifold is nontrivial [29]. Therefore, researchers proposed semi-supervised active learning (SSAL) approaches [30, 31] to learn the representations of input molecules using both labelled and unlabeled data and conductAL on the representation space with the labelled data. However, the available molecules in most public molecular property datasets are still limited (ranging from hundreds to thousands), even without labels.

In this paper, we propose to use molecular representations from a pretrained self-supervised learning model. Specifically, we encoded the molecular SMILES sequences into corresponding embeddings, utilizing a large transformer model MolBERT, pretrained on 1.6 million SMILES via masking, alongside physicochemical properties [20]. The embedding of each SMILES sequence is a pooled output from the pretrained MolBERT with dimension 764. We employed these embeddings from MolBERT to train a fully connected (i.e., MLP) head. This strategy allowed us to leverage a significant volume of molecule data, offering particular benefits for conducting AL on relatively small datasets.

### Practical Bayesian neural networks

In this work, we use a Bayesian neural network to account for the model uncertainty. Previous studies on dropout variational inference [28] suggest that a practical Bayesian neural network for a wide variety of architectures can be obtained by simply training a neural network with dropout (MC dropout), and interpreting this as being equivalent to variational inference [32]. The uncertainty is then estimated by using multiple forward-passes with different dropout masks. Specifically, we conduct 20 stochastic forward passes with dropout rate 0.5, each with a different dropout mask, to obtain a set of predictions. The predictive mean is then calculated by averaging these predictions, and the predictive variance is computed to quantify the model’s uncertainty. Although the uncertainty from MC dropout is often underestimated, it has been a popular choice for Bayesian AL with neural networks and shows promise on real-world datasets [33, 34].

This neural network uses $\varvec{x}_0$ initialized as the input features $\varvec{x}$, which can be either BERT features (in the semi-supervised AL) or binary fingerprints (in the supervised AL). We utilize dropout for uncertainty estimation, batch normalization for training stability, and the rectified linear unit (ReLU) activation function as the default activation. Additionally, the network incorporates a skip connection, merging the input and output of the hidden layer, enhancing information flow. Finally, the output layer generates logits, which can be transformed into probabilities by passing through a sigmoidal activation function.

8 $$ \begin{aligned}&\varvec{x}_0 = \varvec{x}\quad \texttt {BERT features or ECFP}\\&\varvec{x}_{\ell } = \text {Dropout}(\text {ReLU}(\text {BatchNorm}(W_\ell \varvec{x}_0 + \textbf{b}_\ell )))\\&\tilde{\varvec{x}}_{\ell +1} = \text {BatchNorm}(W_{\ell +1} \varvec{x}_{\ell } + \textbf{b}_{\ell +1}) \\&\varvec{x}_{\ell +1} = \text {Dropout}(\text {ReLU}(\varvec{x}_{\ell } + \tilde{\varvec{x}}_{\ell +1}))\\&x_{out} = W_{\ell +2} \varvec{x}_{\ell + 1} + \textbf{b}_{\ell +1} \end{aligned}$$

The hyper-parameters of this model are given in Table 1.

**Table 1:** Hyperparameters used of BNN and training

	Hyperparameter	Values
BNN	Activation	[ReLU]
	Batch normalization	[True]
	Skip connection	[True]
	Input layer	[768, 1024]
	hidden layer dim	[128]
	Number of hidden layers	[1]
	Dropout probability	[0.3]
Training	Optimizer	[Adam]
	Learning rate	[$10^{-3}$]
	Weight decay	[1e-2]
	Scheduler	[CosineAnnealingLR]
	T-max (LR cycle)	[10]
	Batch size	[16]
	Epochs	[110]
	num. Forward pass	[20]

### Baselines

We consider three acquisition functions, random, BALD, and EPIG (Section 2.3), and two learning paradigms, supervised active learning (SAL) and semi-supervised active learning (SSAL). In SSAL, we use the BERT features pretrained on 1.26 million SMILES, and in SAL, we use ECFP, or Extended-Connectivity Fingerprints, directly. ECFP is a method used in cheminformatics to represent molecular structures as binary fingerprints, capturing structural information by encoding the presence or absence of substructural features within a specified radius around each atom. Through iterative traversal of the molecular structure, unique substructural fragments are identified and hashed into a fixed-length bit vector, generating a binary fingerprint where each bit indicates the presence or absence of a specific substructural fragment. We encoded each molecule into a fixed 1024-dimensional binary vector using a radius of 2 (diameter 4)

## Results and discussion

**Fig. 1:**
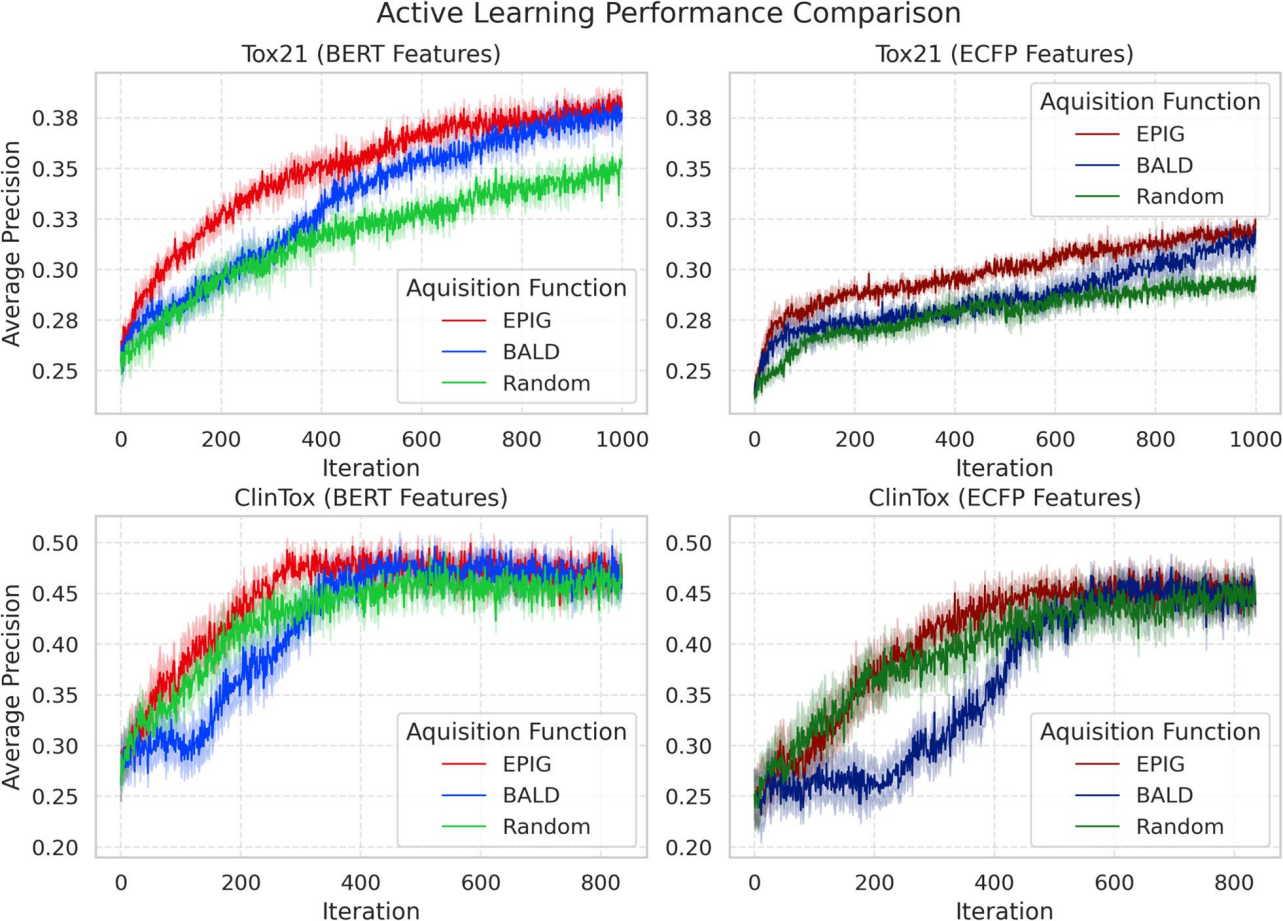
Active learning performance comparison on Tox21 (top) and ClinTox (bottom) datasets using BERT (left) and ECFP (right) molecular representations. BERT features consistently outperform ECFP, with EPIG showing superior sample selection over BALD and Random sampling. Lines show mean performance (averaged across 12 tasks and 3 seeds for Tox21; 10 seeds for ClinTox) with 95% confidence intervals (shaded regions). Evaluation metric is average precision

We evaluated the impact of molecular representations on active learning performance using three acquisition strategies (EPIG, BALD, and Random sampling) on two datasets (Tox21 and ClinTox). For the Tox21 dataset, the impact of feature quality on AL efficiency manifests distinctly across acquisition functions (Fig. 1). BERT-EPIG demonstrates superior learning dynamics with a steeper improvement slope compared to ECFP-EPIG, indicating more efficient sample selection per iteration. The timing of separation from the random baseline highlights the impact of feature quality on uncertainty estimation. BERT-BALD diverges from random sampling, achieving stable significance (p-value < 0.05) at iteration 529. In contrast, ECFP-BALD reaches stable significance only after iteration 878, demonstrating that higher-quality features enable earlier identification of informative samples. Here, stable significance refers to the point after which the performance remains consistently and significantly better (p-value < 0.05) across subsequent iterations.

The ClinTox results further emphasize this pattern while revealing task-specific behaviors (Fig. 1). BERT-EPIG achieves convergence significantly earlier (300 iterations) compared to ECFP-EPIG (600 iterations), demonstrating how high-quality representations accelerate learning. Notably, BALD underperforms random sampling in both feature spaces, aligning with previous findings about BALD’s potential limitations in certain scenarios. These observations, combined with our UMAP visualization showing BERT’s more structured embedding space, strongly support our hypothesis that effective AL fundamentally depends on the quality of molecular representations enabling reliable uncertainty estimation. Statistical validation through Wilcoxon signed-rank tests confirmed BERT-EPIG’s significant superiority over BERT-BALD in both Tox21 (iteration: 300, p-value = $1 \times 10^{-4}$) and in ClinTox (iteration: 300, p-value = $3 \times 10^{-3}$).

### Analysis of learned representations

**Fig. 2:**
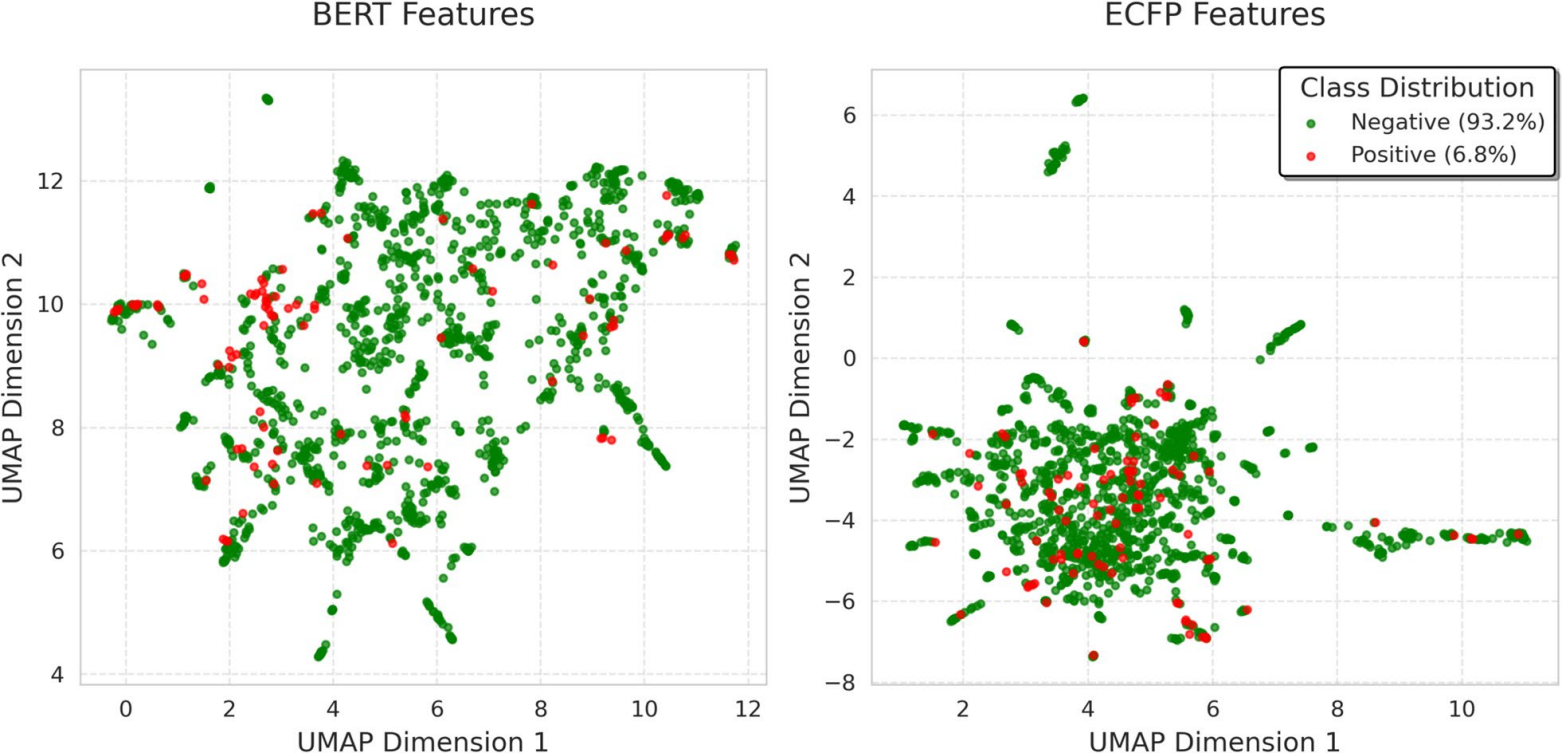
UMAP visualization of molecular features projected into 2D space, BERT (left) and ECFP (right). The points represent individual molecules colored by their class labels (red for positive, green for negative)

To understand why BERT-based approaches significantly outperform ECFP in AL, we visualized both representation spaces using UMAP dimensionality reduction (Fig. 2). The BERT features exhibit more structured organization, where positive samples (red points, 6.8% of dataset) are distributed in distinct clusters, indicating that semantically similar molecules are mapped to nearby regions. This structured manifold enables the model to make better-informed predictions about unlabeled samples based on their proximity to labeled examples, even with limited initial training data. In contrast, ECFP representations show a more scattered distribution with significant overlap between positive and negative regions, making it difficult for the model to learn meaningful patterns from small initial labeled sets. This poorly structured space leads to unreliable uncertainty estimates, explaining why ECFP-based Bayesian acquisition functions (BALD, EPIG) show only marginal improvement over Random sampling.

To address potential concerns about parameter sensitivity in UMAP, we performed a more objective analysis using Principal Component Analysis (PCA) shown in (Appendix Fig. 6). Unlike UMAP, PCA is deterministic and relies on linear projections that maximize variance, providing a parameter-free baseline for comparing representation quality. We further quantitatively evaluated the feature spaces using several complementary metrics: Davies-Bouldin index [35] to measure class separation, class purity to evaluate local sample distributions, and Fisher’s ratio to quantify overall class separability [36]. These metrics collectively demonstrate that BERT’s structural advantages are fundamental rather than artifacts of visualization choices. (supplementary Table 3) These findings support our hypothesis that the effectiveness of uncertainty-based AL methods critically depends on having well-structured molecular representations that enable reliable uncertainty estimation from limited training data.

### Model calibration analysis

**Fig. 3:**
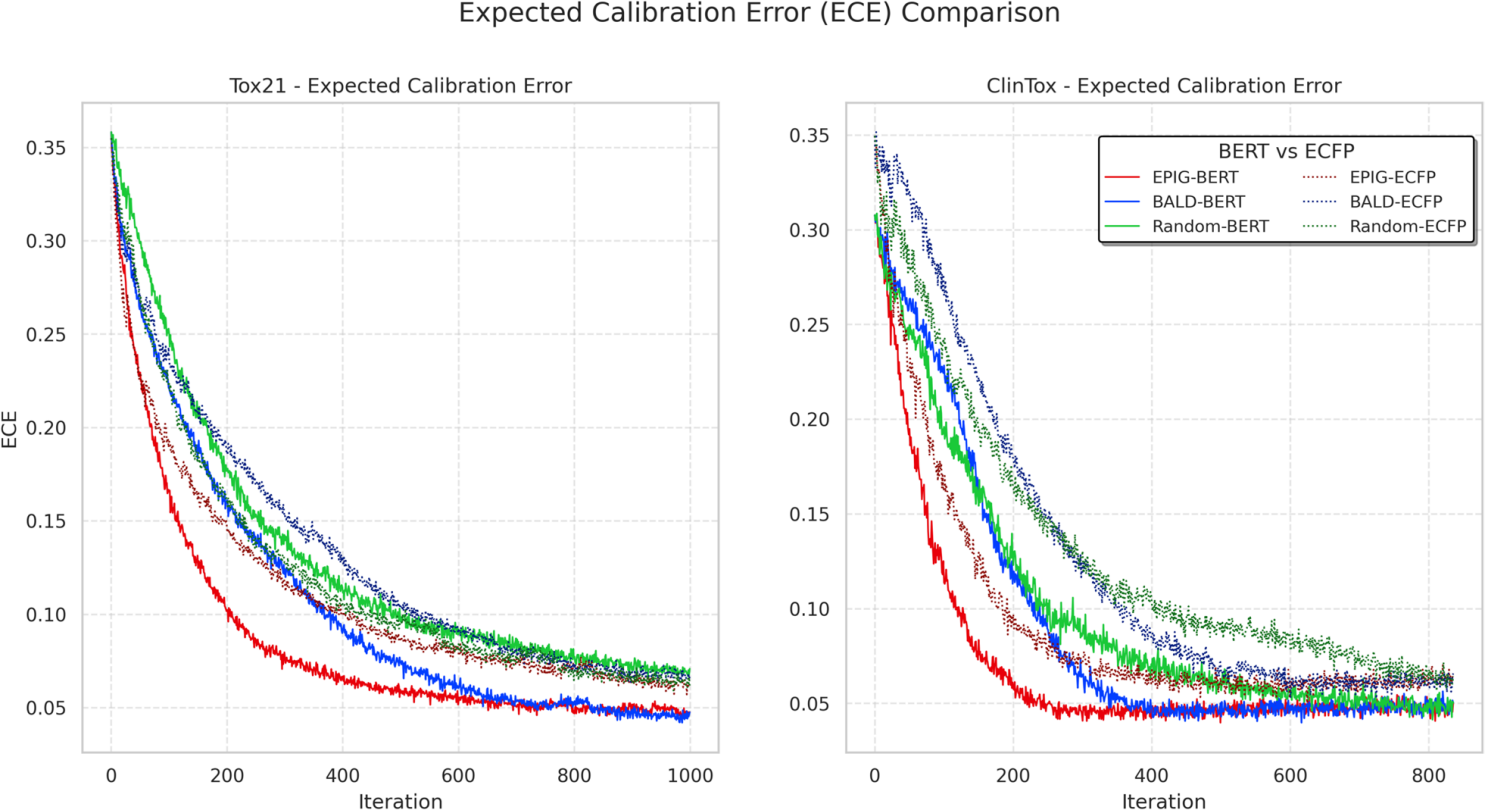
Evolution of Expected Calibration Error (ECE) for Tox21 (left, averaged across 12 tasks and 3 seeds) and ClinTox (right, averaged across 10 seeds). Lower ECE indicates better-calibrated uncertainty estimates. EPIG with BERT features (solid red) achieves the fastest convergence to low ECE values, demonstrating superior uncertainty calibration compared to other methods. While all methods eventually converge to similar ECE values after sufficient iterations, ECFP features require substantially more labeled data to achieve good calibration, highlighting the importance of informative feature representations for reliable uncertainty estimation

To further investigate why Bayesian acquisition functions might underperform with ECFP features, we analyzed the Expected Calibration Error (ECE) by using Eq. 9, throughout the AL process. ECE measures the difference between model confidence and actual accuracy, with lower values indicating better-calibrated uncertainty estimates (13-08-03). Figure 3 shows the evolution of ECE across different feature types and acquisition functions. The results reveal a clear relationship between feature quality and uncertainty estimation. All methods initially exhibit high ECE (0.30$-$0.38), indicating poor calibration due to limited training data. However, the BERT-based approaches demonstrate consistently lower ECE compared to their ECFP counterparts throughout the early stages of AL (iterations 0-200). This aligns with our previous observation that ECFP features lead to less reliable uncertainty estimates, which in turn compromises the effectiveness of Bayesian acquisition functions like BALD and EPIG.

Particularly noteworthy is the EPIG acquisition function with BERT features, which achieves the fastest reduction in ECE (solid red line), suggesting it learns well-calibrated uncertainties more efficiently. This difference in calibration improvement rate is statistically significant when comparing BERT-EPIG to the second-best method, ECFP-BERT, for both Tox21 (iteration 200, p-value = $2 \times 10^{-5}$) and ClinTox (iteration 200, p-value = $4 \times 10^{-4}$). This explains the superior performance of BERT-EPIG, as shown in Fig. 1. In contrast, ECFP-based methods maintain higher ECE for a longer period, indicating persistent struggles in uncertainty estimation despite sophisticated acquisition strategies.

While all methods eventually converge to well-calibrated uncertainties (ECE < 0.1) after 600-800 iterations, the path to achieving good calibration is markedly different. ECFP-based approaches require substantially more labeled data to achieve comparable calibration, which is particularly problematic in the AL setting where labeled data is initially scarce. This finding reinforces our hypothesis that the success of Bayesian acquisition functions is fundamentally limited by the quality of input representations and their ability to enable reliable uncertainty estimation from limited training data.

### Analysis of sample acquisition patterns

**Fig. 4:**
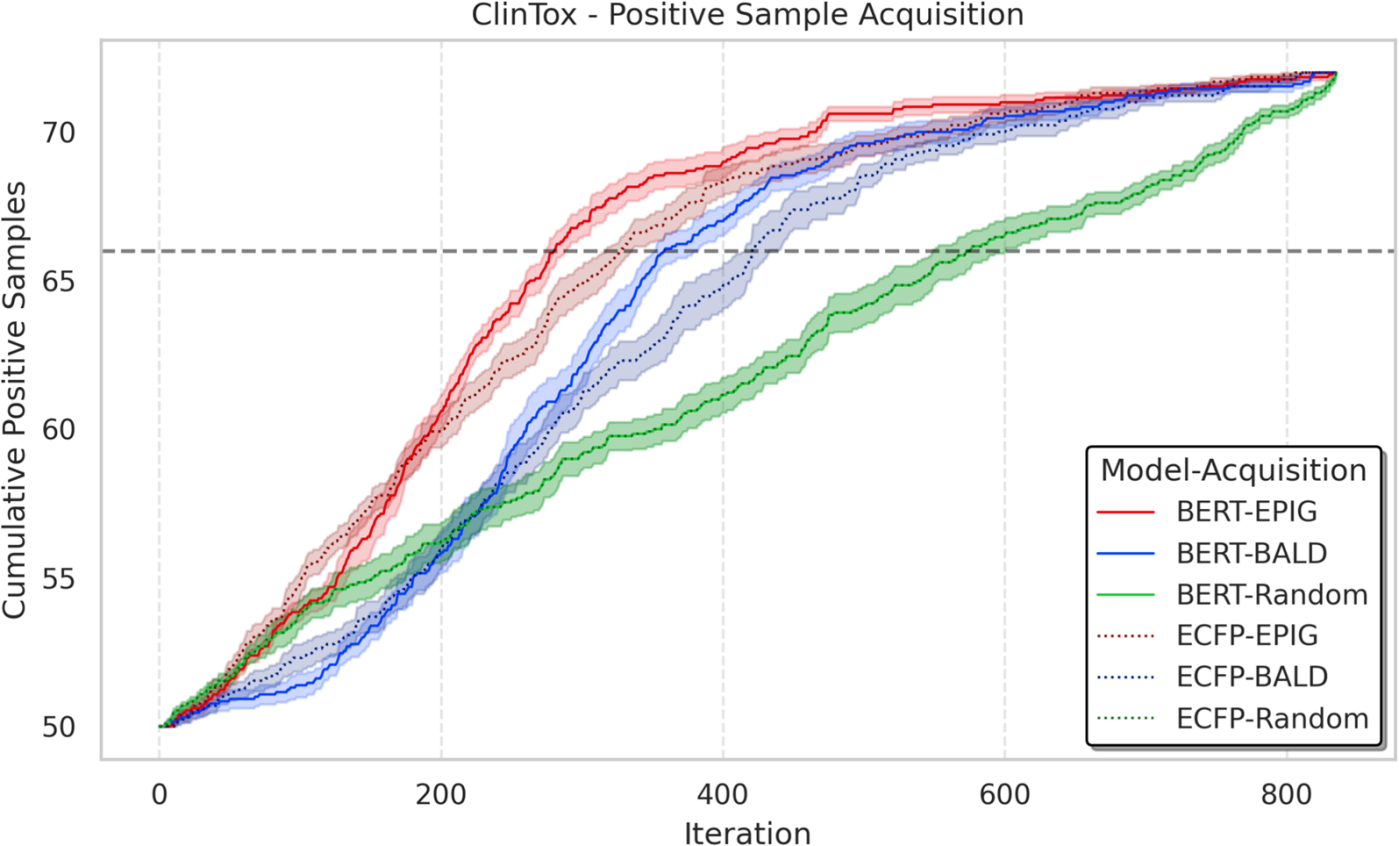
Comparison of positive sample acquisition rates across different feature representations and acquisition functions on the ClinTox dataset , where mean and standard error is computed across 10 seeds. The plot shows cumulative toxic compound identification starting from a balanced initial set (50 positive, 50 negative). BERT-EPIG demonstrated a 2-fold improvement over Random sampling by identifying 70% of toxic compounds (gray horizontal line) in only 266 iterations compared to approximately 600 iterations for Random sampling, demonstrating better exploration of the chemical space when starting with limited labeled data

To further understand why BERT representations enable more effective AL, we analyzed the cumulative acquisition of positive samples (toxic compounds) across iterations (Fig. 4). Starting from a balanced initial set (50 positive, 50 negative samples), the acquisition patterns reveal key differences between BERT and ECFP approaches in handling the significant class imbalance present in the pool set (22 positive out of 835 samples).

BERT-EPIG demonstrates superior acquisition efficiency, identifying 70% of toxic compounds in only 266 iterations, compared to 343 iterations for BERT-BALD and approximately 600 iterations for Random sampling. BERT-EPIG demonstrated a 2-fold improvement over Random sampling in identifying toxic compounds. This accelerated discovery of minority class samples aligns with the structured representation space observed in UMAP visualization (Fig. 2), where BERT features organize molecules into meaningful clusters that facilitate identification of informative toxic compounds.

Interestingly, while ECFP-EPIG initially shows comparable acquisition rates to BERT-EPIG, its performance plateaus earlier, suggesting that the scattered representation space limits its ability to make reliable uncertainty estimates as learning progresses. We further confirmed the statistical superiority of BERT-EPIG over ECFP-EPIG in positive sample selection through a Wilcoxon signed-rank test (at iteration 300, p-value = $5 \times 10^{-3}$). ECFP-BALD exhibits similar limitations, highlighting that even sophisticated Bayesian acquisition functions struggle when the underlying representation space lacks clear structure for learning from limited initial data.

### Better features enable better uncertainty estimation

**Fig. 5:**
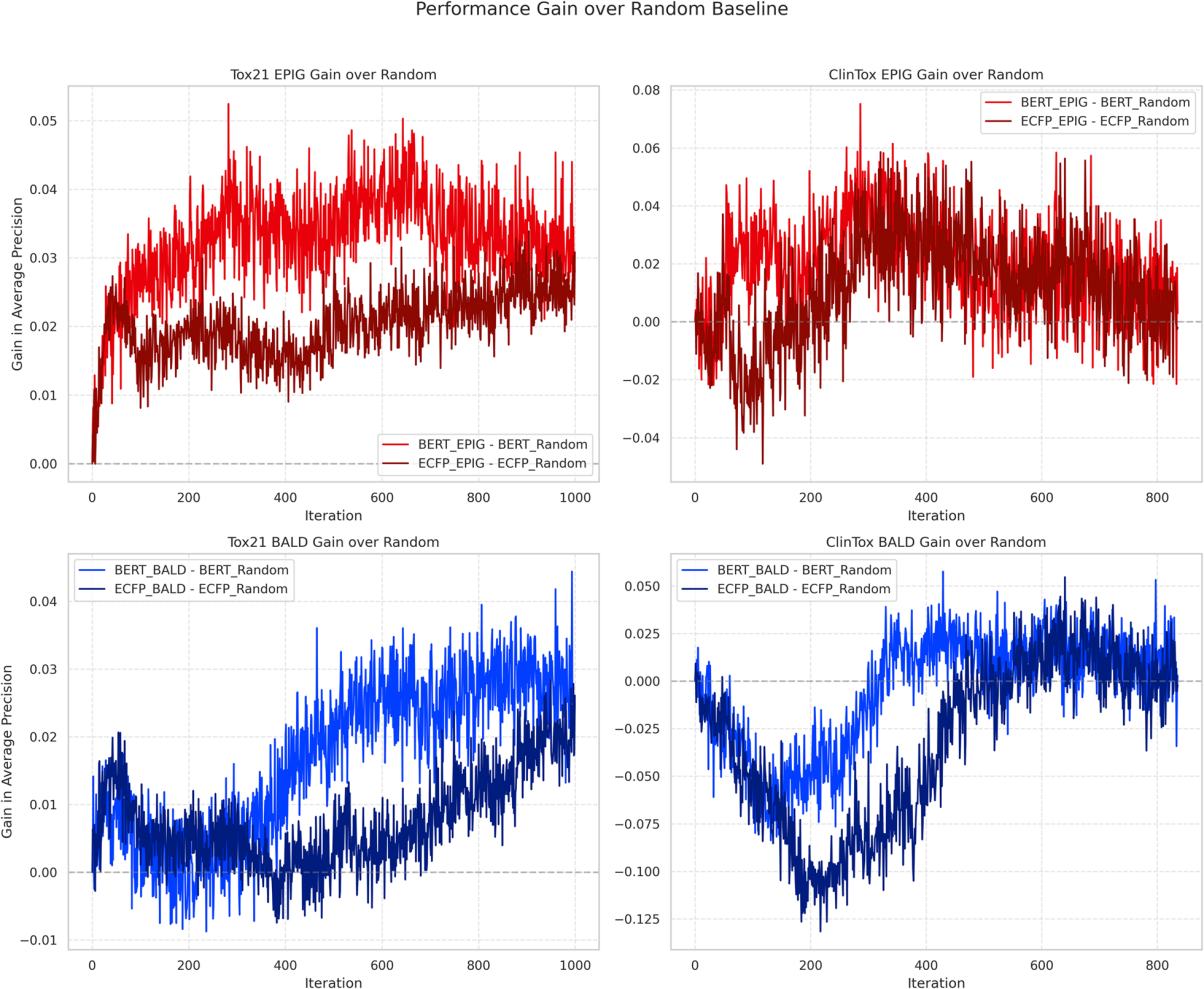
Performance gains of EPIG (top) and BALD (bottom) compared to random sampling baseline for Tox21 (left) and ClinTox (right). BERT features (light colors) show consistently higher gains than ECFP (dark colors), with EPIG demonstrating more stable improvements than BALD across iterations. The y-axis shows the difference in average precision between each acquisition function and its corresponding random baseline (averaged across 12 tasks and 3 seeds for Tox21; 10 seeds for ClinTox)

Our experimental results reveal two key aspects of AL performance: absolute gains from feature representations and relative gains from acquisition functions. Comparing absolute performance (Fig. 1), BERT features consistently outperform ECFP, with BERT-EPIG achieving the highest average precision (0.38 for Tox21, 0.50 for ClinTox). While this superior performance could stem from better feature quality, we demonstrate it primarily arises from improved uncertainty estimation.

To disentangle these factors, we analyzed relative gains over random sampling baselines (Fig. 5). The steeper slope of BERT-EPIG’s gain curve in early iterations (0-200) indicates more accurate uncertainty estimation, leading to efficient sample acquisition. In Tox21, BERT-EPIG achieves a maximum gain of 0.05 over BERT-Random, compared to ECFP-EPIG’s 0.02 gain over ECFP-Random (at iteration 282, p-value = $1 \times 10^{-3}$). This disparity suggests BERT features not only provide better base performance but also enable more reliable uncertainty estimation for superior sample selection.

The acquisition function comparison further reveals EPIG’s advantages over BALD. While BALD shows positive gains after 400-600 iterations, EPIG maintains consistent improvements from early stages. This difference is most pronounced in ClinTox, where ECFP-BALD initially degrades performance ($-$0.125) before recovery, while EPIG maintains stable gains. These findings demonstrate that successful molecular property prediction requires both high-quality representations and well-calibrated uncertainty estimation, with BERT-EPIG optimally combining both aspects.

## Conclusion

Our study demonstrates that the success of AL in molecular property prediction depends critically on the synergy between feature representations and acquisition functions. BERT features enable more effective uncertainty estimation compared to ECFP, as evidenced by faster ECE convergence and steeper learning curves. EPIG consistently outperforms BALD, maintaining stable improvements from early iterations across both datasets.

The superior performance of BERT-EPIG stems from two key factors: (1) BERT’s structured representation space, which clusters chemically similar compounds, facilitating reliable uncertainty estimation from limited data, and (2) EPIG’s ability to leverage this structure for efficient sample acquisition, particularly in identifying rare positive samples. This combination achieves up to 0.05 and 0.08 improvements in average precision over random sampling for Tox21 and ClinTox, respectively.

These findings highlight that successful AL requires both high-quality molecular representations and well-calibrated uncertainty estimation. Future work in molecular property prediction should focus on developing feature representations that enable reliable uncertainty quantification, particularly in low-data regimes.

## Figures and Tables

**Fig. 6:**
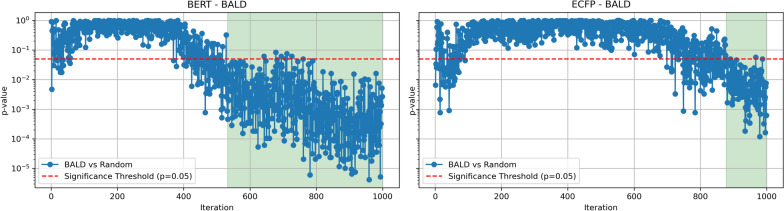
Statistical significance of BALD acquisition function compared to random sampling in Tox21 dataset. The plots show p-values (log scale) for the performance difference between BALD and random sampling acquisition strategies across training iterations for BERT (left) and ECFP (right) molecular representations. The red dashed line indicates the significance threshold (p=0.05), with values below this line representing statistically significant improvements. Green shaded regions highlight where BALD consistently achieves statistical significance. BERT representations show earlier and more pronounced statistical significance (beginning around iteration 500) compared to ECFP, which only shows consistent significance much later (around iteration 850)

**Fig. 7:**
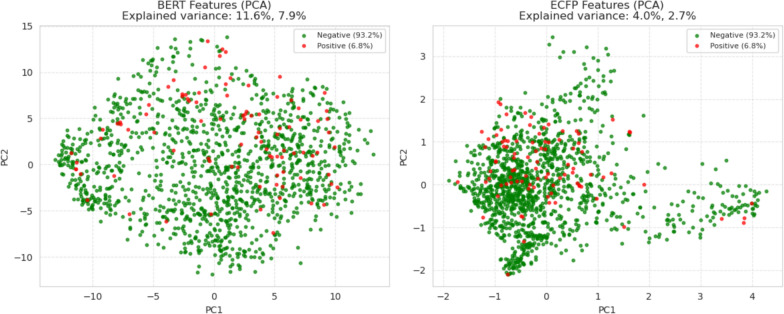
PCA projection of molecular representations. **a** BERT embeddings showing more coherent clustering of positive samples (red). **b** ECFP embeddings with less distinct separation between classes

**Fig. 8:**
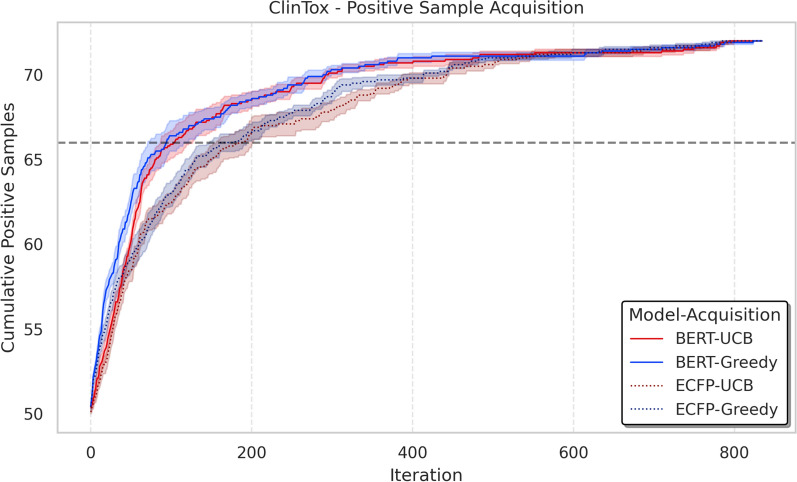
Cumulative positive sample acquisition in ClinTox dataset using BERT and ECFP features with UCB and Greedy acquisition functions. Mean and standard error computed across 10 random seeds. Starting from a balanced initial set (50 positive, 50 negative), BERT-based models extracted 70% of positive samples in $\sim 100$ iterations, compared to $\sim 180$ iterations for ECFP-based models—a nearly twofold difference in sample acquisition efficiency

**Fig. 9:**
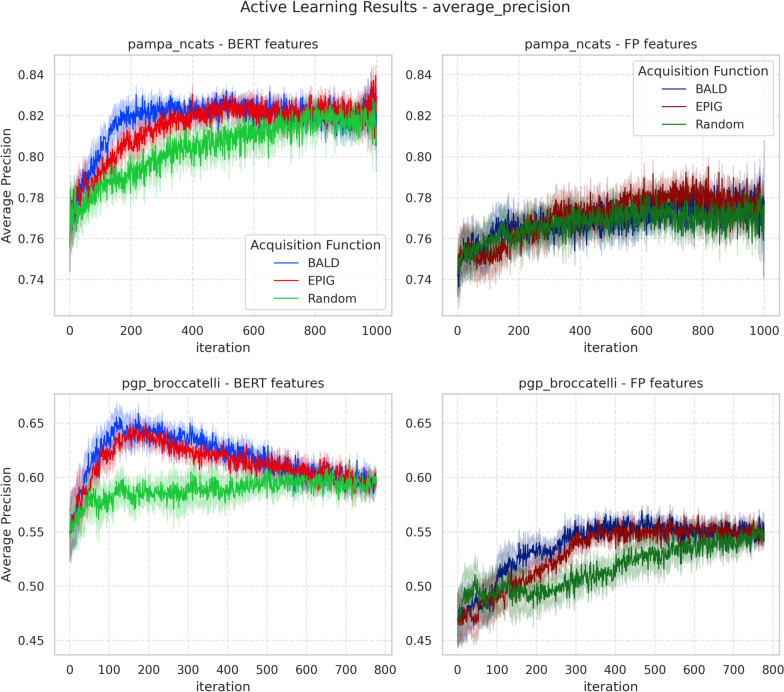
Active learning performance comparison across PAMPA permeability and Pgp inhibition datasets using BERT and ECFP features

**Fig. 10:**
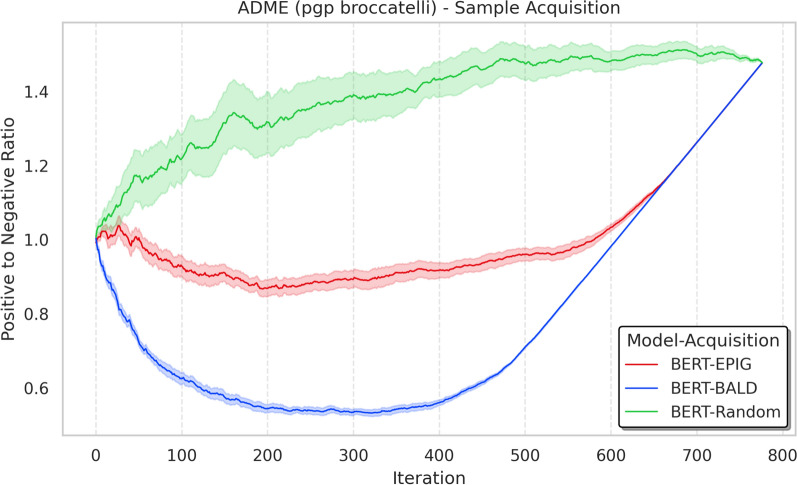
Positive-to-negative sample ratio during active learning for the Pgp broccatelli dataset using different acquisition strategies with BERT representations

## Data Availability

We will upload the code, datasets, acquired samples, and results in project GitHub repository (https://github.com/Arslan-Masood/Active-learning-with-BERT).

## A Appendix

See Table 2.

**Table 2:** Dataset statistics showing the distribution of positive (active) and negative (inactive) samples across different sets

	Total			Initial Set			Pool Set			Val Set			Test Set		
Dataset	Pos	Neg	Pos/Neg	Pos	Neg	Pos/Neg	Pos	Neg	Pos/Neg	Pos	Neg	Pos/Neg	Pos	Neg	Pos/Neg
Tox21															
NR-AR	299	6704	0.04	50	50	1.0	156	4262	0.04	39	1066	0.04	54	1326	0.04
NR-AR-LBD	222	6321	0.04	50	50	1.0	106	4084	0.03	27	1020	0.03	39	1167	0.03
NR-AhR	764	5558	0.14	50	50	1.0	428	3593	0.12	107	898	0.12	179	1017	0.18
NR-Aromatase	286	5333	0.05	50	50	1.0	118	3511	0.03	30	877	0.03	88	895	0.1
NR-ER	766	5233	0.15	50	50	1.0	462	3404	0.14	116	850	0.14	138	929	0.15
NR-ER-LBD	320	6395	0.05	50	50	1.0	178	4100	0.04	45	1025	0.04	47	1220	0.04
NR-PPAR-gamma	164	6083	0.03	50	50	1.0	50	3977	0.01	13	994	0.01	51	1062	0.05
SR-ARE	900	4802	0.19	50	50	1.0	505	3213	0.16	126	803	0.16	219	736	0.3
SR-ATAD5	243	6599	0.04	50	50	1.0	103	4260	0.02	26	1065	0.02	64	1224	0.05
SR-HSE	336	5971	0.06	50	50	1.0	156	3896	0.04	39	974	0.04	91	1051	0.09
SR-MMP	871	4732	0.18	50	50	1.0	498	3117	0.16	125	779	0.16	198	786	0.25
SR-p53	384	6161	0.06	50	50	1.0	159	4013	0.04	40	1003	0.04	135	1095	0.12
ClinTox	92	1261	0.073	50	50	1.0	22	813	0.027	4	143	0.028	16	255	0.063
pampa ncats	1737	289	6.01	25	25	1.0	1142	114	10.02	285	29	9.83	285	121	2.36
hia hou	497	69	7.2	25	25	1.0	286	36	7.94	72	8	9.0	114	0	–
pgp broccatelli	645	561	1.15	25	25	1.0	446	285	1.56	111	72	1.54	63	179	0.35

Train and test sets are created using an 80/20 scaffold split, with the training set further divided into initial set, validation set, and pool set through random splitting. All reported results in plots are based on the test set.

### A.1 Results and discussion

The statistical significance analysis of BALD acquisition with BERT and ECFP features is shown in Fig. 6.

### A.2 Quantitative analysis of molecular representations

To objectively evaluate the structural differences between BERT and ECFP representations, we performed a comprehensive quantitative assessment directly on the original high-dimensional feature spaces. This approach provides an unbiased view of the inherent properties of these molecular representations without the potential artifacts introduced by dimensionality reduction techniques. Table 3 presents the quantitative metrics comparing BERT and ECFP representations in their native feature spaces.

These quantitative results provide strong evidence supporting our original claims about the superiority of BERT representations for active learning in toxicity prediction. The Davies-Bouldin index, where lower values indicate better cluster separation, shows that BERT (6.046) provides 36.5% better separation between toxic and non-toxic compounds compared to ECFP (9.529). This improved separation is critical for active learning methods that rely on clear decision boundaries for uncertainty estimation.

For the minority positive class (toxic compounds), BERT embeddings demonstrate 69.5% higher neighborhood purity (0.154 vs. 0.091), indicating that toxic compounds are more likely to be grouped together rather than scattered throughout the representation space. This characteristic is particularly valuable in imbalanced classification scenarios like toxicity prediction, where finding rare positive samples is the primary challenge. While both representations show high negative class purity, BERT still maintains a slight advantage (0.961 vs. 0.955), suggesting better cohesion of the majority negative class as well.

Finally, Fisher’s ratio-a direct measure of class separability that quantifies the ratio of between-class to within-class variance-is 2.5 times higher for BERT (0.054) compared to ECFP (0.021). This 153% improvement provides an objective confirmation that BERT representations inherently separate toxic from non-toxic compounds more effectively even in their original high-dimensional space.

These quantitative metrics offer objective evidence that the structural advantages of BERT representations are fundamental rather than artifacts of specific dimensionality reduction or visualization choices.

**Table 3:** Comparison of BERT and ECFP Feature Representations

Metric	BERT	ECFP	Description
Davies-Bouldin score	6.046	9.529	Measures ratio of within-cluster scatter to between-cluster separation; lower is better
Positive class purity	0.154	0.091	Average fraction of positive samples in neighborhoods around positive samples
Negative class purity	0.961	0.955	Average fraction of negative samples in neighborhoods around negative samples
Fisher’s ratio	0.054	0.021	Ratio of between-class to within-class variance; higher values indicate better class separation

### A.3 Model calibration analysis

Expected Calibration Error (ECE) measures how closely a model’s predictive confidence aligns with its actual accuracy by binning predictions and calculating the weighted average of absolute differences between bin accuracy and confidence (Fig. 7).

9 $$\begin{aligned} \text {ECE} = \sum _{m=1}^{M} \frac{|B_m|}{n} \left| \text {acc}(B_m) - \text {conf}(B_m) \right| \end{aligned}$$

where *M* is the number of bins, $B_m$ is the set of predictions in bin *m*, $|B_m|$ is the number of predictions in bin *m*, *n* is the total number of predictions, $\text {acc}(B_m)$ is the accuracy of predictions in bin *m*, and $\text {conf}(B_m)$ is the average predicted confidence of samples in bin *m*.

### A.4 Analysis of sample acquisition patterns

Here we compare the cumulative positive sample acquisition for the ClinTox dataset using two acquisition functions commonly employed in Bayesian optimization frameworks. The Upper Confidence Bound (UCB) acquisition function $\text {UCB}(x) = \mu (x) + \beta \sigma (x)$ balances exploration and exploitation by combining the predicted model performance $\mu (x)$ with its uncertainty $\sigma (x)$, using a fixed exploration parameter $\beta = 2$. In contrast, the Greedy acquisition function $\text {Greedy}(x) = \mu (x)$ simply selects points based on the highest predicted mean, without explicitly considering uncertainty. BERT-based models efficiently extracted 70% of positive samples in approximately 100 iterations, while ECFP-based models required around 180 iterations—nearly a twofold increase in sample acquisition effort. However, no significant differences were observed between the Greedy and UCB acquisition functions across both feature representations. One possible reason might be that UCB requires further fine-tuning of $\beta$, which is outside the scope of this study (Fig. 8).

### A.5 ADME datasets

Additionally, to demonstrate broader applicability, we selected the first three classification datasets from the TDC ADME benchmark (PAMPA Permeability, Human Intestinal Absorption, and Pgp Inhibition). We had to drop second dataset as scaffold split (80/20) yielded no positive samples in the test set. Figure 9 demonstrates that the performance advantages observed in toxicity prediction extend to ADME properties. For PAMPA permeability (top row), both BALD and EPIG acquisition functions with BERT representations achieve higher average precision compared to Random sampling. Statistical analysis confirms these observations, with both BERT-BALD and BERT-EPIG showing significant improvements over Random sampling(BERT-BALD vs. Random: $p = 9 \times 10^{-6}$; BERT-EPIG vs. Random: $p = 2 \times 10^{-3}$; iteration = 200). In contrast, when using ECFP features for the same dataset, both BALD and EPIG perform nearly identically to Random sampling throughout the entire learning process.

For Pgp inhibition (bottom row), we observe a similar pattern. With BERT representations, both BALD and EPIG quickly separate from Random sampling (around iteration 50-100). Conversely, with ECFP features, the separation from random sampling occurs much later (around iteration 250-300) and the performance gap remains considerably smaller throughout the learning process.

Both EPIG and BALD with BERT features show decline in average precision after peaking at iterations 150-200. This decline stems from a distribution mismatch-the pool set has more positives (ratio 1.56) while the test set has more negatives (ratio 0.35). Figure 10 shows uncertainty-based methods preferentially select minority negative samples from the pool, with BERT-BALD’s positive-to-negative ratio dropping from 1.0 to 0.55 by iteration 300. This sampling initially improves performance but eventually creates harmful distribution shift.

This difference in acquisition function behavior between representation types demonstrates that BERT’s structural properties enable more effective uncertainty estimation, while also highlighting how the interplay between acquisition strategies and dataset distributions can significantly impact active learning outcomes [37].
